# Synergetic Effect of Electrical and Topographical Cues in Aniline Trimer-Based Polyurethane Fibrous Scaffolds on Tissue Regeneration

**DOI:** 10.3390/jfb14040185

**Published:** 2023-03-28

**Authors:** Yinglong Zhang, Jiajing Tang, Wei Fang, Qing Zhao, Xiaoyu Lei, Jinzheng Zhang, Jieqiong Chen, Yubao Li, Yi Zuo

**Affiliations:** 1 Research Center for Nano-Biomaterials, Analytical and Testing Center, Sichuan University, Chengdu 610064, China; 2 MOE Key Laboratory of Low-Grade Energy, Utilization Technologies and Systems, CQU-NUS Renewable, Energy Materials & Devices Joint Laboratory, School of Energy & Power Engineering, Chongqing University, Chongqing 400044, China

**Keywords:** oligoaniline, conductive polyurethane, fibrous scaffolds, electrical and topographical cues, tissue regeneration

## Abstract

Processibility and biodegradability of conductive polymers are major concerns when they are applied to tissue regeneration. This study synthesizes dissolvable and conductive aniline trimer-based polyurethane copolymers (DCPU) and processes them into scaffolds by using electrospinning with different patterns (random, oriented, and latticed). The effects of topographic cue changes on electrical signal transmission and further regulation of cell behaviors concerning bone tissue are researched. Results show that DCPU fibrous scaffolds possessed good hydrophilicity, swelling capacity, elasticity, and fast biodegradability in enzymatic liquid. In addition, the conductivity and efficiency of electrical signal transmission can be tuned by changing the surface’s topological structure. Among them, oriented DCPU scaffolds (DCPU-O) showed the best conductivity with the lowest ionic resistance value. Furthermore, the viability and proliferation results of bone mesenchymal stem cells (BMSCs) demonstrate a significant increase on three DCPU scaffolds compared to AT-free scaffolds (DPU-R). Especially, DCPU-O scaffolds exhibit superior abilities to promote cell proliferation because of their unique surface topography and excellent electroactivity. Concurrently, the DCPU-O scaffolds can synergistically promote osteogenic differentiation in terms of osteogenic differentiation and gene expression levels when combined with electrical stimulation. Together, these results suggest a promising use of DCPU-O fibrous scaffolds in the application of tissue regeneration.

## 1. Introduction

When tissues are injured, various biological signals are changed to promote the natural regeneration process, such as electrical potential gradient, chemical gradients, and mechanical stress [1,2,3]. Among these, endogenous electrical signals induced by wounds have proven to be the priority signals guiding cell migration and promoting tissue repair [4,5]. Diverse tissues including the nervous system, heart, bone, muscle, and skin present electrically sensitive properties in the human body, of which many functions are regulated by these endogenous signals [6,7,8,9,10,11]. Studies have shown that healthy bone tissue can generate electrical signals to maintain its physiological homeostasis by activating voltage-controlled calcium channels in the plasma membrane [12]. However, damaged periosteum is prone to interrupt the transmission of electrical signals that will apparently slow down the healing of bone tissue [13,14]. Therefore, smart substitutes based on conductive biomaterials are in urgent need for the rebuilding of severe periosteal injury.

Compared to traditional insulation biomaterials, conductive polymers (CPs), such as polyaniline (PANi), polypyrrole (PPy), and polythiophene (PEDOT) have shown great potential in biological stimulation by charge transferring in situ [15,16,17]. Unfortunately, most of these conductive polymers are non-degradable or poorly biodegradable, which may cause an immune response after long-term implantation [18,19,20,21]. Worse still, due to indissoluble conjugated segments, poor processibility of these CPs mounts a serious challenge to fabricate specific morphology or a complex structure suitable for tissue repairing the field [22]. 

Efforts have been taken to develop biodegradable substrates through blending conductive components with degradable polymers [23,24]. However, in the few biodegradable scaffolds, CPs might split off from the mixtures due to the weak binding force between the blends, leading to inflammation [25,26]. Recently, oligoaniline-based CPs have shown great potential in solving the drawbacks because studies have demonstrated that oligomers can be phagocytosed by macrophages without triggering an immune response [27,28]. Different from the conjugated CPs, oligoaniline could provide designed polymers sufficient electroactivity with good biodegradability [29,30]. Moreover, polyurethane (PU) as widely used biomaterials with excellent biocompatibility and flexibility, can be designed to achieve the desired properties by adjusting the components and proportions of soft and hard segments [11,31]. Accordingly, different oligoaniline-based PUs have been reported, such as electroactive and biodegradable PU based on polycaprolactone (PCL), polyethylene glycol (PEG), and aniline pentamer (AP) for cardiac tissue engineering applications [32]. Shape memory PU elastomers based on poly (citric acid-co-polycaprolactone) (CA-PCL), dopamine (DA), and aniline hexamer (AH) for skeletal muscle regeneration [16]. Nevertheless, the prepared oligoaniline-based polymers are still limited in subsequent processability. 

Moreover, the topologic structure of biomaterials is another vital aspect that affects tissue regeneration, which mediates the biological response between the materials and the host tissue directly. Cell response induced by topographic cues of biomaterials has been reported [33,34,35]. Wang et al. proved that aligned polyester fibers as topographic cues could enhance osteogenic differentiation by reducing the peroxisome proliferator-activated receptor gamma (PPARγ) signaling pathway [36]. Previous reports have also revealed that aligned morphologies of various electroactive substrates can greatly influence the behavior of neural cells [37]. Hence, it is expected that the synergetic effect of electrical and topographic cues will provide an efficient way to accelerate the repairing of bone tissue.

In this study, we aimed to fabricate conductive biomaterials with biodegradability and processability, which could be processed into scaffolds with both electrical and topographic cues. Hence, we successfully synthesized easily dissolved conductive polyurethane (DCPU) functionalized with aniline trimer-co-_L_-Lysine, of which the DCPU copolymer has better dissolvability than the electroactive polyurethane gel (PUAT) reported before [38]. Whereafter, a series of DCPU fibrous scaffolds with three patterns (random, oriented, and latticed) as topographic cues were prepared by using the electrospinning technique. Concomitantly, aniline trimer (AT) was chosen as the electrical cue through the polymerization of DCPU while _L_-Lysine was selected to further improve polymer degradation except for the adoption of degradable soft segments (PCL). Herein, the molecular structures, surface topological structure, thermal properties, hydrophilicity, swelling capacity, mechanical properties, conductivity, and biodegradability of DCPU fibrous scaffolds were investigated. On this premise, the beneficial effect of DCPU fibrous scaffolds by topographic and electrical cues on BMSCs was evaluated to explore its potential application in bone tissue engineering.

## 2. Materials and Methods

### 2.1. Materials

Poly(ε-caprolactone)-diol (PCL-diol, *Mn* = 2000), Isophorone diisocyanate (IPDI), _L_-Lysine, aniline, p-phenylenediamine, stannous salt, and N-Methylformamide were purchased from the Aladdin Co., Ltd. of Shanghai, China. Hydrochloric acid (HCl), ammonium persulfate, dimethyl sulfoxide (DMSO), N, N-Dimethylformamide (DMF), aqueous ammonia (NH_3_∙H_2_O), and ethanol were obtained from Kelong Co., Ltd. of Chengdu, China. PCL-diol, _L_-Lysine, N-Methylformamide, DMSO, and DMF were dried by the activated molecular sieve before use.

### 2.2. Synthesis of AT

The synthesis of amine-capped AT was similar to a previous report [39]. In brief, p-phenylenediamine was dissolved in a three-neck flask containing a mixed solution of 1 M HCl and 1 M ethanol (*V*:*V* = 5:2). The solution was stirred and cooled in a circulating cooling pump. Ammonium persulfate was added into the mixed solution after the temperature was under −5 °C. Subsequently, the distilled aniline was added rapidly into the reaction solution to avoid the overoxidation of p-phenylenediamine. After the reaction, the precipitate was filtered by a Buchner funnel and washed using HCl followed by distilled water. The product was dedoped and then filtrated. The obtained AT was washed using distilled water and then dried by lyophilization. After that, a rotary evaporator was used to purify the AT. The product was ground using agate mortar and then dried by lyophilizer.

### 2.3. Synthesis of DPU and DCPU Copolymers

The prepolymers were prepared using IPDI as hard segments, PCL as soft segments, and stannous salt as the catalyst in a three-neck flask full of nitrogen atmosphere. After stirring at 75 °C for hours pre-polymerization, _L_-Lysine dispersed in DMSO solution was then added as a chain extender and reacted at 50 °C for another 2 h. Subsequently, the electroactive AT dissolved in anhydrous DMF was added and stirred at room temperature till the reaction was terminated. The copolymers were precipitated in methanol and washed with deionized water. The obtained polymerization products were vacuum-dried for 48 h. The copolymers with and without electroactive AT were named as follows: (1) DPU, dissolved polyurethane, the molar ratio of PCL/IPDI/_L_-Lysine/AT was 1:2:1:0; (2) DCPU, dissolved and conductive polyurethane, the molar ratio of PCL/IPDI/_L_-Lysine/AT was 1:2:0.5:0.5.

### 2.4. Preparation of Scaffolds with Different Patterns

The DPU and DCPU copolymers were dissolved in HFIP to form a 20 *w*/*v* % solution, respectively. Scaffolds with different topological patterns were fabricated by different electrospinning collecting devices. The plate receiver, the rotating cylinder, and the reseau receiver were used to collect the fibers to obtain different scaffolds with random, oriented, and latticed surface topology. The flow rate of the solution was 0.5 mL/h. A voltage of 10 kV and 20 cm distance was applied between the needle and the collector. When using the rotating cylinder, the rotating speed was 2800 rpm. The DPU scaffold with random surface topology was used as the control. Samples were named as DPU-R (DPU scaffold with random fibers), DCPU-R (DCPU scaffold with random fibers), DCPU-O (DCPU scaffold with orientated fibers), and DCPU-L (DCPU scaffold with latticed fibers). All collected fibrous scaffolds were placed in a vacuum drying oven for 3 days to eliminate solvent adequately.

### 2.5. Physicochemical Characterizations of Copolymers

Proton nuclear magnetic resonance (^1^H NMR) spectroscopy, fourier transform infrared (FT-IR) spectroscopy, and gel permeation chromatography (GPC) were used to investigate the chemical and physical characterizations of DPU and DCPU copolymers. Moreover, the thermal physical properties of the copolymers, including decomposition temperature (*T_d_*) and glass transition temperature (*T_g_*), were also measured. The crystalline structures of the copolymers were studied by X-ray diffraction analysis. The electroactivity of DCPU copolymer was tested by cyclic voltammetry (CV) and UV−vis spectroscopy. The testing details are available in Materials and Methods, Supporting Information.

### 2.6. Dissolvability and Processibility Testing

Different solvents were used in the solubility experiments to test the dissolvability of DPU and DCPU copolymers. The electroactive PU organogel (PUAT) prepared according to our previous study was used as the control [38]. The stable processibility of DPU and DCPU copolymers has been verified through electrospinning to fabricate fibrous scaffolds with three patterns (random, oriented, and latticed). The morphology of DCPU fibrous scaffolds was observed by scanning electron microscopy (SEM; JSM-7500F, JEOL, Tokyo, Japan). The diameter of the fibers was processed by Image-Pro Plus software.

### 2.7. Electroactivity of DCPU Copolymer

The electroactivity of DCPU copolymer was investigated by the UV-vis spectroscopy and cyclic voltammetry (CV). The UV-vis spectroscopy of AT, DPU, and DCPU copolymers were recorded on an UV−vis spectrophotometer (PerkinElmer Lambda 35) with the solution of DMF. Cyclic voltammograms (CV) were recorded on an electrochemical workstation (CHI 660E, China) with a scanning rate of 10 mV/s in 1 M HCl solution. A platinum plate, and an Ag/AgCl electrode were used as counter and reference electrodes, respectively. A graphite rod was immersed in the DCPU solution and dried in the oven. The DCPU copolymer was coated on the graphite rod and employed as working electrode.

### 2.8. Physicochemical Performance of Different Scaffolds

The hydrophilicity of scaffolds was measured by water contact angle measurements (JY-82B, Chengdu, China). The swelling capacity of the scaffolds was studied by testing the water uptake profile of the scaffolds in PBS (pH = 7.2–7.4) at 37 °C. The mechanical properties of the DPU and DCPU scaffolds were evaluated by the uniaxial tensile test and cyclic tensile test employing a universal testing machine (SHIMADZU, AG-IC 50KN, Tokyo, Japan). The conductivity and electrochemical properties of DCPU scaffolds were tested with a digital 4-probe tester (Keithley 6517A, Cleveland, OH, USA) and an electrochemical workstation (CHI 600E, CH Instruments, Shanghai, China), respectively. The testing details are available in Materials and Methods, Supporting Information.

### 2.9. In Vitro Biodegradability, Cytocompatibility and Differentiation Effected by Scaffolds

#### 2.9.1. In Vitro Enzymatic Degradation

The biodegradability of different scaffolds was tested with an in vitro enzyme-accelerated degradation experiment. Briefly, the scaffolds were cut into specimens (10 × 10 mm^2^, ~200 μm thick) and immersed in 4 mL PBS containing 300 U/mL lipase from Burkholdria cepacian at 37 °C. At regular time intervals, the buffer solution was renewed, and samples were removed, washed twice with distilled water, and freeze dried to a constant weight before weighing. The residual weight was calculated as:

Weight Loss (%) = *W*_3_ − *W*_2_/*W*_3_ × 100% (1)

where *W*_3_ and *W*_2_ represent the weights of scaffolds before and after degradation, respectively.

#### 2.9.2. In Vitro Cytocompatibility

Cell proliferation and live cell assay were performed to investigate the cytocompatibility of the fibrous scaffolds. All the fibrous scaffolds were irradiated by ^60^Co (15 kGy) to sterilize before the experiment. Sprague-Dawley (SD) rats were obtained from Lilai Experimental Animals Co., Ltd. (Chengdu, China), and BMSCs were extracted from the femurs and tibiae of the SD rats weighing about 100 g. Biological experiments were conducted in accordance with the guidelines approved by the Animal Ethics Committee of Sichuan University (Protocol No. 20211534A). The BMSCs were incubated with α-MEM (Gibco, Grand Island, NE, USA), 10% newborn calf serum (NBCS, Gibco, USA), and a 1% mixture of penicillin/streptomycin (MP Biomedicals, Santa Ana, CA, USA) in an incubator at 37 °C with flowing air containing 5% CO_2_. The scaffolds were incubated in a cell culture medium for 12 h before seeding. The BMSCs were seeded onto the DPU and DCPU fibrous scaffolds in 12-well culture plates with the density of 1.5 × 10^4^ cells/well.

The proliferation of BMSCs cultured on different surface topological fibrous scaffolds was assessed by the cell counting kit-8 (Sigma-Aldrich Co., St. Louis, MO, USA) on days 1, 4, and 7. The blank group without any materials was used as the control group, and each group was prepared in triplicate. The Multilabel Counter (Wallac Victor 31420, PerkinElmer Co., Waltham, MA, USA) was performed to measure the absorption value at 450 nm. Live BMSCs fluorescence staining was performed after the samples were washed with PBS three times. Calcein AM (0.25 μM) (KeyGEN BioTECH, Jiangsu, China) was added to the samples for 45 min. Whereafter, cells were observed by an inverted fluorescence microscope (IX53, Olympus).

#### 2.9.3. Cells Morphology

To further investigate the impact of the diverse surface topological structure nanofiber matrix on BMSCs’ growth geometric, the cells cultured on the fibrous scaffolds were stained. First, 1 × 10^4^ BMSCs were cultured into every well in a 12-well plate, and the growth medium was discarded after incubation for 5 days. Second, 4% formaldehyde solution was added to fix the cells for 30 min and then they were cleaned with PBS to eliminate excessive formaldehyde. Third, cells’ F-actin and nucleus were labelled by Alexa Fluor^®^ 546 phalloidin and Hoechst 33342, respectively. In the last step, fluorescence images of the cellular morphology were obtained with a confocal laser scanning microscope (CLSM; AIR, MP+, Nikon, Tokyo, Japan). Image-Pro Plus software was used to quantify the cell nuclei orientation angle, nucleus area, and the cell nucleus aspect ratio. Mean values and standard deviations from 100 randomly chosen cells were calculated.

#### 2.9.4. MSCs Differentiation under Electrical Stimulation

To further discover the influence of electroactive fibrous scaffolds on the osteogenic differentiation of BMSCs, electrical stimulation (ES) was conducted on the groups of control and DCPU-O scaffolds by using an electromagnetic coil launcher which can produce a low-frequency pulsed electromagnetic field (Jiuneng, China) shown in Figure S1. An electric potential was applied by placing the sample into a magnetic induction coil. The parameters of ES were set at a square wave, frequency of 15 Hz, electrical potential of 700 mV, 100% duty cycle and 30 min per day according to references [40,41]. ES was adopted after cells were seeded on the samples for 12 h. In the following experiment, BMSCs were cultivated under ES and without ES. After 7 days of cultivation, the expressions of four of the osteogenesis-related genes, including alkaline phosphatase (ALP), runt-related transcription factor 2 (Runx 2), osteocalcin (OCN) and osteopontin (OPN) were assessed by using the real-time PCR technique following the instructions.

The cells were digested from the samples, and the RNA of the samples was extracted using the Animal Total RNA Isolation Kit. The reverse transcription was performed on a PCR instrument using the All-In-One Mastermix (with AccuRT Genomic DNA Removal kit). When the cDNAs were generated, the real-time PCR analysis was conducted using the Stepone plus Real-time PCR System (Applied Biosystems Inc., Waltham, MA, USA) and a certain protocol. The gene expression was quantified using SYBR Premix Ex Taq RR420A. Gene-specific primers, including glyceraldehyde-3-phosphate dehydrogenase (GAPDH), ALP, Runx 2, OCN, and OPN were designed and shown in Table S1. The gene expression levels were obtained using the threshold cycles (*C*_t_). Relative transcript quantities were calculated by the ΔΔ*C*_t_ method. GAPDH was used as a reference gene and amplified together with the target genes from the same cDNA samples. The difference in the C_t_ value of the sample relative to GAPDH was defined as Δ*C*_t_. The difference between the ΔC_t_ of the experimental group cells and Δ*C*_t_ of the cells grown in the control group was defined as the ΔΔC_t_. The fold change in gene expression was expressed as 2^-ΔΔCt^.

### 2.10. Statistical Analysis

All of the data from the study were presented as the mean ± standard deviation. One-way analysis of variance (ANOVA) tests, together with a post-hoc Tukey’s test or Dunnett’s multiple comparisons test was conducted using the SPSS (version 25.0) software (LEAD Technologies, Inc., Chicago, IL, USA) to examine the data by the following significance levels: * *p* < 0.05, ** *p* < 0.01, and *** *p* < 0.001, which represent statistically significant, very significant, and extremely significant values, respectively. *p* > 0.05 indicated that there were no statistically significant values.

## 3. Results and Discussion

### 3.1. Synthesis of AT

The molecular structure of produced AT was investigated by ^1^H NMR and MS spectra. As shown in Figure S2 (Supporting Information), the peaks at 6.95 ppm (s, 2H, Ar H), 6.78 ppm (s, 2H, Ar H), and 6.61 ppm (s, 2H, Ar H) were related to the aromatic protons of AT structures [15,42]. Moreover, peaks at 5.42 ppm (s, 4H, NH_2_) and 4.60 ppm (s, 4H, NH_2_) were corresponding to the amine groups in the oxidation state and the reduction state, respectively [43]. Interestingly, there was no obvious peak around 5.42 ppm when the AT was changed to the fully reduced state by hydrazine hydrate. This result indicated that the synthesized AT was the mixture of the oxidation state and the reduction state. Furthermore, the charge-mass ratio tested by mass spectrometry indicated that the molecular weight of AT was 288 g/mol (Figure S3) [44]. The results confirmed that the electroactive AT was successfully synthesized.

### 3.2. Synthesis of Conductive Copolymer

**Figure 1:**
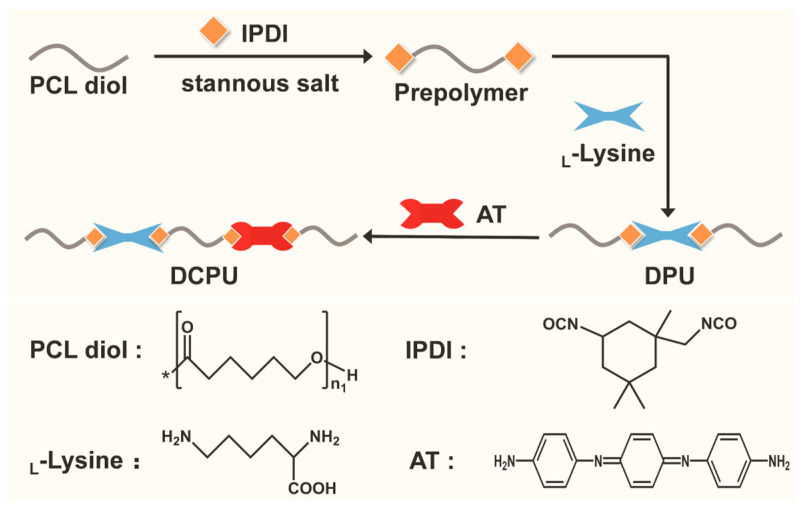
Synthesis route of DPU and DCPU copolymers (*: the repeat unit of PCL-diol).

**Figure 2:**
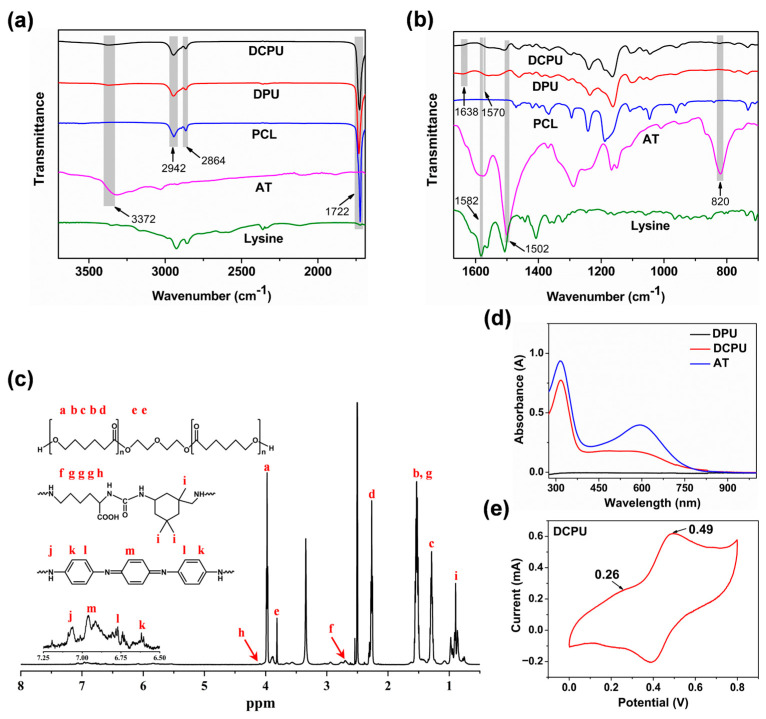
Physicochemical characterizations of DPU and DCPU copolymers: (**a**,**b**) FT-IR spectra of the DPU and DCPU copolymers and their raw materials in different wave band, (**c**) ^1^H NMR spectrum of the DCPU copolymer (the different letters represented the correspondence between chemical structure of materials and peaks in the ^1^H NMR), (**d**) UV−vis spectra of AT, DPU, and DCPU dispersed in DMF, (**e**) Cyclic voltammogram of DCPU copolymer in 1 M HCl.

As shown in Figure 1, the copolymers were synthesized by melting polycondensation of PCL-diol, IPDI, AT, and _L_-Lysine. Figure 2a,b showed different characteristics of FT-IR spectra of the raw materials, DPU, and DCPU copolymers in a different wave band. For the characteristic absorption of −NCO groups, no absorption peak was observed at 2260 cm^−1^ (N=C stretching) in the DCPU curves, indicating that all −NCO groups were totally consumed in the copolymerized synthesis. Accordingly, the characteristic peak appeared at 3372 cm^−1^ attributed to the vibrational bands of urethane and urea groups. The amine group absorption peaks at 3450 cm^−1^, 3320 cm^−1^, and 3209 cm^−1^ in AT were converted into a single absorption peak at 3372 cm^−1^ in DCPU curves, indicating the successful transformation of amine groups from AT into urea units. In addition, the peaks at 2942 cm^−1^ and 2864 cm^−1^ were attributed to methylene groups from PCL, and the peak at 1722 cm^−1^ was assigned to the carbonyl group (C=O) in the ester bond. The peak at 1570 cm^−1^ in DPU and DCPU copolymers, in comparison with the peak at 1582 cm^−1^ of _L_-Lysine in the infrared spectrum, generated a red shift of about 12 cm^−1^, which was due to the reduction of adsorption force of carboxyl ion [45]. Furthermore, the peaks at 1502 and 820 cm^−1^ were corresponding to the vibrational benzenoid ring, and 1, 4 replace the benzenoid ring from AT, respectively. Moreover, there was a new peak at 1638 cm^−1^ in DPU and DCPU copolymers, corresponding to the asymmetric stretching absorption peak of the carbonyl group for urea linkages, revealing the transformation of −NH_2_ groups in AT and _L_-Lysine into urea groups after reacting with IPDI [46]. 

The molecular structure of the DCPU copolymer was testified by ^1^H NMR. As shown in Figure 2c, peaks at 3.98 ppm (t, 2H, CH_2_OH), 3.81 ppm (s, 2H, CH_2_O), 2.26 ppm (t, 2H, CH_2_), 1.56–1.52 ppm (m, 2 × 2H, CH_2_) and 1.29 ppm (m, 2H, CH_2_) were attributed to PCL segments [17]. The peaks between 0.86 and 0.98 ppm (m, 3H, CH_3_) correspond to the IPDI segments, while peaks at 4.10 ppm (m, H, CH) and 2.80–2.67 ppm (m, 2H, CH_2_N) correspond to _L_-Lysine segments. The peak at 7.06 ppm (s, 2H, NH), 6.95 ppm (s, 2H, Ar H), 6.78 ppm (s, 2H, Ar H), and 6.61 ppm (s, 2H, Ar H) were related to the aromatic protons of AT structures as well as to urethane and urea N–H bonds [32]. Wherein, the produced AT was investigated by ^1^H NMR (Figure S2). In addition, there was no prominent peak around 5.42 ppm and 4.60 ppm, indicating that the amine groups (NH_2_) of AT were successfully reacted with isocyanate groups (N=C=O) and transformed into urea groups appearing at 7.06 ppm. The results of FT-IR and ^1^H NMR demonstrated that the AT and _L_-Lysine were successfully connected to the DCPU backbone. Furthermore, the molecular weight and polydispersity index of DCPU copolymer increased with the addition of AT compared to DPU (Table S2). All the data from FT-IR, NMR, and GPC demonstrated the successful synthesis of DPU and DCPU copolymers.

The electroactivity of DCPU copolymer was evaluated using UV−vis spectroscopy measurements and cyclic voltammetry (CV). Figure 2d showed the UV-visible spectra of AT, DPU, and DCPU copolymers. The peak at about 315 and 597 nm of pure AT were presented, on account of the π−π* transition of the benzene ring and the benzenoid-to-quinoid excitonic transition, respectively [16]. Moreover, the lower wavelength absorption peaks at 590 nm of the DCPU copolymer compared to the AT sample were attributed to the formation of electron-withdrawing urea linkage, which significantly reduced the electron density of AT segment [47,48]. However, there were no such absorption peaks in DPU copolymer due to the lack of electroactive AT. The electroactivity of the DCPU copolymer was further demonstrated by CV measurements. Aniline oligomers have different oxidation states, which named the leucoemeraldine state (LM), the emeraldine state (EM) and the pernigraniline state (PN), respectively. All of the three states can be reversibly changed when reacted with different voltages or oxidating/reducing agents [49]. As shown in Figure 2e, the DCPU copolymer presented two pairs of well-defined reduction/oxidation peaks at about 0.26 and 0.49 V, corresponding to the transitions from the LM state to the EM state and the EM state to the PN state, respectively (Figure S4d). Both the UV−vis spectroscopy and CV results demonstrated the good electroactivity of the DCPU copolymer. According to the measured TGA and DSC analysis, the thermal stability of DCPU copolymer was improved with the AT segment, which the decomposition temperature has largely increased with DCPU copolymer (Figure S4a,b). In addition, the result of XRD analysis confirmed the crystallinity of the copolymer decreased (Figure S4c), revealing there were more hard segments dispersed in the soft phase and more robust interaction between the polymer chains in DCPU copolymer. This might be the reason why the glass transition temperature (*T_g_*) of DCPU was higher than that of DPU copolymer.

### 3.3. Dissolvability, Processibility and Topological Structure of Scaffolds

**Figure 3:**
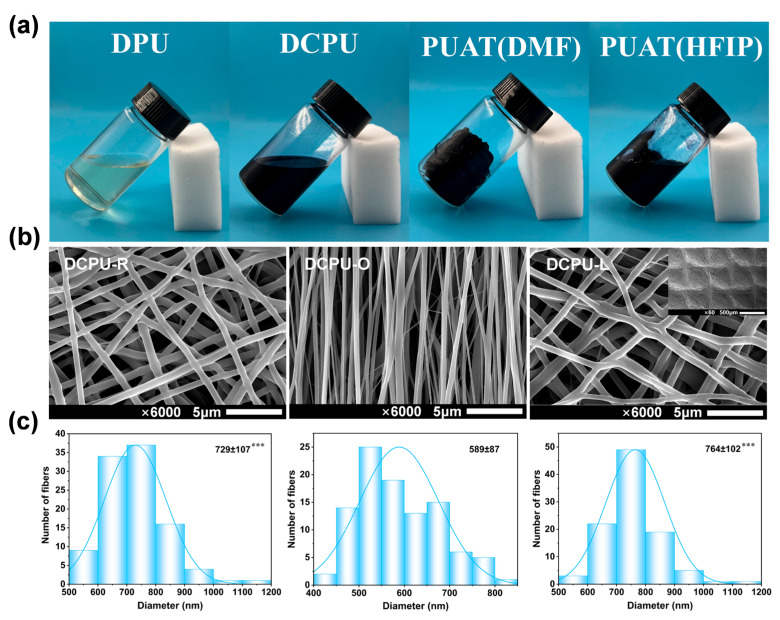
Dissolvability, processibility of copolymers and topological structure of scaffolds: (**a**) Digital photos of the dissolution state of copolymers in solvents; (**b**) Surface topological structure of DCPU fibrous scaffolds; (**c**) Fibers’ diameter of fibrous networks with three different surface topological structure. *** *p* < 0.001 statistically significant compared with DCPU-O (*n* = 100).

The application of most conductive polymers has been inhibited by poor processibility. To explore the possibility for processing, different solvents were used in the solubility experiments to test the dissolvability of DPU and DCPU copolymers. In the process of dissolution, polymeric scaffolds imbibe solvent molecules at first, forming a swollen polymeric gel. Once the swelling reaches a critical point, the polymer chains start disentangling from the swollen polymer gel and eventually disperse into the solution [50]. Our control material, i.e., the PUAT organogel, tended to swell instead of dissolving in the solvents even if DCPU and PUAT were prepared at the same raw material ratio (Table 1 and Figure 3a), so that it was difficult to accurately shape except for cast molding. As shown in Table 1 and Figure 3a, DPU and DCPU copolymers were soluble in the majority of common solvents to meet the requirement of electrospinning, whereas the PUAT organogel was poorly soluble in these solvents. Furthermore, the GPC results showed that the molecular weight of DPU, DCPU copolymers, and PUAT organogel were in the same order of magnitude range (Table S2), therefore the solubility was attributable to the nature of the copolymer itself. In this work, the amino-terminal group of _L_-Lysine as the chain extender had reacted with the prepolymer before the addition of AT, which was different to the simultaneous feed of AT and _L_-Lysine in the polymerization of PUAT organogel. The change of adding order of raw materials resulted in the isocyanate group being partly consumed by _L_-Lysine before reacting with AT. In addition, the reaction activity of AT and the isocyanate group were depressed by lowing the reaction temperature and stirring speed, which led to less entanglement of the polymer segment in DPU and DCPU copolymers. Overall, due to the change of synthesis conditions, the resulting DCPU copolymers exhibited larger molecular weight and better solubility than the PUAT organogel.

After the successful synthesis of the DCPU copolymers, they were processed into three different topological fibrous scaffolds by using electrospinning. The SEM images (Figure 3b) and diameter distribution (Figure 3c) showed that the DCPU fibers were arranged disordered, oriented, and latticed in the three scaffolds, respectively. The filaments in the DCPU-R scaffold were randomly distributed, the filaments in the DCPU-O scaffold were aligned and closely arranged with each other, and the filaments in the DCPU-L scaffold were interwoven to form a grid of small cells, of which the mesh diameter was about 400 μm. Compared with the filaments in DCPU-R and DCPU-L scaffold, the filaments in the DCPU-O scaffold showed the smallest diameter around 589 nm (*p* < 0.001 to DCPU-R and DCPU-L), which was caused by the stretching of the fibrous as the receiver rotates during electrospinning [51].

### 3.4. Physicochemical Performance of Different Scaffolds

The physicochemical properties of DPU and DCPU fibrous scaffolds including hydrophilicity, swelling capacity, and mechanical properties were systematically explored. Research indicated that the water contact angle of 40° to 90° could enhance the cell attachment on the surface of implanted biomaterials efficiently [52]. The water contact angle values of different fibrous scaffolds did not exceed this range, indicating that these fibrous membranes might have good cell attachment (Figure S5a, Supporting Information). Interestingly, the DCPU-O scaffolds showed the lowest water contact angle of 63° in contrast to other AT-added scaffolds (*p* < 0.01 to DCPU-R and *p* < 0.001 to DCPU-L, respectively), which indicated the best hydrophilia of DCPU-O scaffolds. 

The DCPU-R and DCPU-O scaffolds showed slightly lower swelling capacity compared to the DPU-R scaffold in early contact with water for its hydrophobic benzene ring of the added AT segment. However, DCPU-L scaffolds showed the best swelling capacity compared with other groups (Figure S5b, Supporting Information). This phenomenon was due to the highest specific surface area and large number of uneven grid cells on the surface of DCPU-L fibrous scaffolds (Figure 3b and Table S3). The swelling capacity of the conductive fibrous scaffolds varied with the AT segment ratio and/or the topological surface structure, indicating these fibrous scaffolds exhibited tunable swelling capacity. 

The tensile stress−strain curves and the mechanical properties of the fibrous scaffolds were shown in Figure S5c and Table S4. The tensile strength and initial modulus of fibrous scaffolds increased significantly with the addition of AT compared to the pristine DPU-R scaffolds. (Table S4) Moreover, the DCPU-O scaffold showed the highest tensile strength (32.37 ± 4.08 MPa, *p* < 0.05 versus DCPU-R and DCPU-L) and initial modulus (13.41 ± 2.39 MPa, *p* < 0.05 versus DCPU-R and *p* < 0.01 versus DCPU-L) due to their well-aligned arrangement among all scaffolds. This revealed that the AT segment ratio and the topography were the main factors affecting the tensile strength. The elongation at the break of all fibrous scaffolds was greater than 400%, and small irreversible deformations occurred at a maximum strain of 200%, indicating that these fibrous scaffolds all had good tensile properties and could be adapted to a variety of biological applications (Figure S5d and Table S4, Supporting Information).

### 3.5. Electrochemical Performances of Scaffolds

Considering the wet state of the fibrous scaffolds during cell culture and in vivo application, the conductivity of three DCPU fibrous scaffolds with different surface topological structures was tested under both dry and wet states, and the results are shown in Table 2. For the dry state, DCPU-O fibrous scaffolds showed the highest conductivity with the value of 7.15 ± 0.49 × 10^−10^ S/cm, about 1.78 times and 2.13 times over than that of DCPU-R and DCPU-L scaffold, respectively. It was worth noting that the conductivity of DCPU scaffolds in the dry state was increased at least 1.24-fold higher than that in the conductive polyurethane casting membranes reported before (2.7 ± 0.9 × 10^−10^ S/cm), though the feed ratio of the AT component was doubled in the casting membrane [53]. The difference of conductivity between fibrous scaffolds and casting membranes in the dry state might be due to the structure of materials. In other words, the fibers in DCPU scaffolds could lead to a faster charge transport in contrast to the solidified copolymer in the casting membranes. After being immersed in PBS, all three kinds of scaffolds showed dramatically increased conductivity, ranging from 1.89 ± 0.15 × 10^−5^ to 4.09 ± 0.51 × 10^−5^ S/cm. The conductivities of wet DCPU fibrous scaffolds were in the range of semiconductor materials (~10^−8^ − 1000 S/cm), which were similar to those of human physiological environments, revealing their potential applications for bone tissue engineering [54]. Significantly, the conductivity of fibrous scaffolds in the wet state increased sharply about four orders of magnitude compared to that in the dry state. The difference of casting membranes’ conductivity between the dry and wet state were just two orders of magnitude [55,56]. These results were due to the high surface area-to-volume ratio and porosity in the fibrous scaffolds (Table S3), which could absorb more liquid and ion when immersed in the PBS. Interestingly, the conductivity of DCPU-O fibrous scaffold was about twice that of DCPU-R and DCPU-L, both dry and wet states. The difference of conductivity was attributed to different charge transport channels caused by the fibers’ distribution and topologic structure [57]. This phenomenon will be explored in detail in the next experiment.

**Figure 4:**
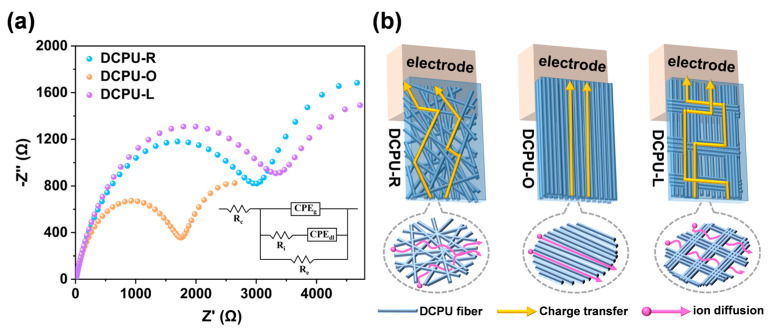
Electrochemical properties of the DCPU fibrous networks: (**a**) Nyquist plot and equivalent circuit model obtained from the EIS characterization of fibrous networks with three patterns, (**b**) schematic illustration of the charge transfer and ion diffusion pathways through fibrous networks with three patterns.

On account of the electrochemical impedance spectroscopy (EIS) being used in the field of electrochemistry to monitor the effect of conductive polymers on electron and ion transport, we performed the EIS test on the three different topological fibrous scaffolds to investigate their electrical properties further [58]. Figure 4a demonstrated the Nyquist plots for all three DCPU scaffolds and the high frequency region revealed a semicircle. Interestingly, the Nyquist plot of the DCPU-O scaffold showed a smaller semicircle, indicating a lower charge transfer resistance and more efficient electrolyte ion diffusion when compared with the DCPU-R and DCPU-L scaffolds [59]. In order to better understand the electrochemical behavior of the scaffolds, the Nyquist plots with typical equivalent circuits have been fitted, and the specific values for the different circuit elements are summarized in Table 3. *R*_e_ represented the electronic resistance, *R*_i_ represented the ionic resistance, *R*_c_ represented the total ohmic resistance of the electrochemical cell assembly, and *CPE*_dl_ and *CPE*_g_ represented the constant phase elements (*CPE*) corresponding to the double-layer ionic capacitance and the geometric capacitance, respectively [60,61]. Compared with the DCPU-R and DCPU-L scaffolds, DCPU-O scaffold showed the lowest ionic and electronic resistance values (*R*_e_), about half of the other two groups. The results of EIS were consistent with the conductivity of three different DCPU scaffolds tested before. The major factor on the different conductivity between three DCPU scaffolds was the path of electrical signal transmission caused by filaments’ distribution instead of water uptake ability. Even the water uptake ratio of the DCPU-L fibrous scaffolds was significantly higher than that of the DCPU-R and DCPU-O scaffolds (Figure S5b). To be specific, scaffolds with aligned filaments were free of crossing junctions that formed in aggregated reseaux, or wire-to-wire junctions in random distributed scaffolds (Figure 4b). The charge transfer and ion diffusion would be less impeded between oriented fibers, similar to a previous study [57]. In a word, the DCPU-O scaffolds could construct a conductive channel with higher efficiency to enhance the communication between materials and electrical signals. All data from the conductivity and EIS demonstrated the best electrical signal transfer properties of the DCPU-O fibrous scaffold in contrast to the DCPU-R and DCPU-L scaffolds.

### 3.6. In Vitro Biodegradability, Cytocompatibility and Differentiation Effected by Scaffolds

#### 3.6.1. In Vitro Biodegradability

**Figure 5:**
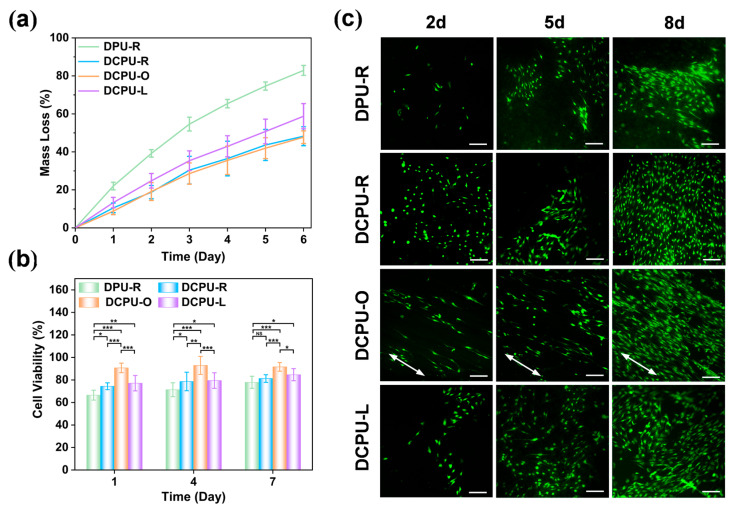
In vitro biodegradability and cytocompatibility of fibrous scaffolds: (**a**) in vitro degradation profiles immersed in lipase solution (*n* = 5); (**b**) CCK-8 analysis of BMSCs cultured on DPU-R and DCPU fibrous scaffolds on day 1, 4, and 7 days (* *p* < 0.05, ** *p* < 0.01, *** *p* < 0.001) (*n* = 3); (**c**) Live cell fluorescence staining images of BMSCs seeded on DPU-R and DCPU fibrous scaffolds on day 2, 5, and 8 days. Scale bar: 100 μm. Arrows: cell orientation.

Biodegradability is the critical requirement of bone regeneration [36]. The results of in vitro enzymatic degradation are shown in Figure 5a. The DPU-R fibrous scaffold showed the fastest degradation rate, and above 80% of the mass was degraded within 6 days. The degradation rates of the DCPU fibrous scaffolds with three different patterns were slower than that of DPU-R, caused by the higher molecular weight and stronger interaction between the polymer chains of DCPU with the addition of AT segment. Among the three groups with different topological surfaces, the group of DCPU-L fibrous scaffolds showed a relatively fast degradation rate. Nearly 60% of the mass was degraded within 6 days, due to more lipase solvent taken part in the degradation while the abundant uneven grid unit could absorb more liquid inside the polymer matrix (better swelling capacity of DCPU-L in Figure S5b). Nevertheless, on account of no significant difference in composition and lack of an uneven grid unit in the groups of DCPU-R and DCPU-O, they exhibited similar rates of degradation. The DCPU fibrous scaffolds presented fast degradation in a high enzymatic solution (300 U/mL) within 6 days, which would be suitable for long-term tissue regeneration in vivo where the lipase concentration under normal physiological conditions is only 0.03~0.19 U/mL [62].

#### 3.6.2. In Vitro Cytocompatibility

In bone tissue engineering, an electroactive substrate is postulated to facilitate the bone regeneration. Electroactive materials have a positive effect on bone tissue that could promote the attachment, proliferation, and differentiation of osteoblasts [9]. Herein, CCK-8 assay was used to evaluate the cell viability. As shown in Figure 5b, the cell viability of three DCPU groups (DCPU-R, DCPU-O and DCPU-L) was significantly higher than that of the DPU-R group at all time points, indicating better cytocompatibility of the DCPU groups. Notably, the DCPU-R group exhibited higher cell viability than the DPU-R group with the same topologic structure (*p* < 0.05). This result suggested that the addition of the electroactive AT segment could promote the proliferation of BMSCs. Among the three DCPU groups, the cell viability of the DCPU-O group was maintained steadily at around 92% at all time points, which was significantly higher than that of other two groups (*p* < 0.05). Meanwhile, the cell viability of the DCPU-R group and the DCPU-L group was at a similar level. This result revealed that the oriented topological structure could significantly enhance cell proliferation.

In addition, live cell staining was also performed to verify the results of cell viability. As shown in Figure 5c, the number of MSCs in each group increased with the culture time. Three DCPU groups (DCPU-R, DCPU-O and DCPU-L) had more cells than the AT-free DPU-R group, especially on day 8, which was consistent with the results of CCK-8 assay. Interestingly, the cells in the DCPU-O group followed the same direction (arrows), while the cells in the other groups were random. This phenomenon will be explored in detail in the next experiment. The above results showed that the electrical and topographic cues significantly affected cell proliferation. The electroactive DCPU scaffolds exhibited better biocompatibility and cell proliferation ability than the non-electroactive DPU materials, especially the DCPU-O fibrous scaffolds which proliferated rapidly.

**Figure 6:**
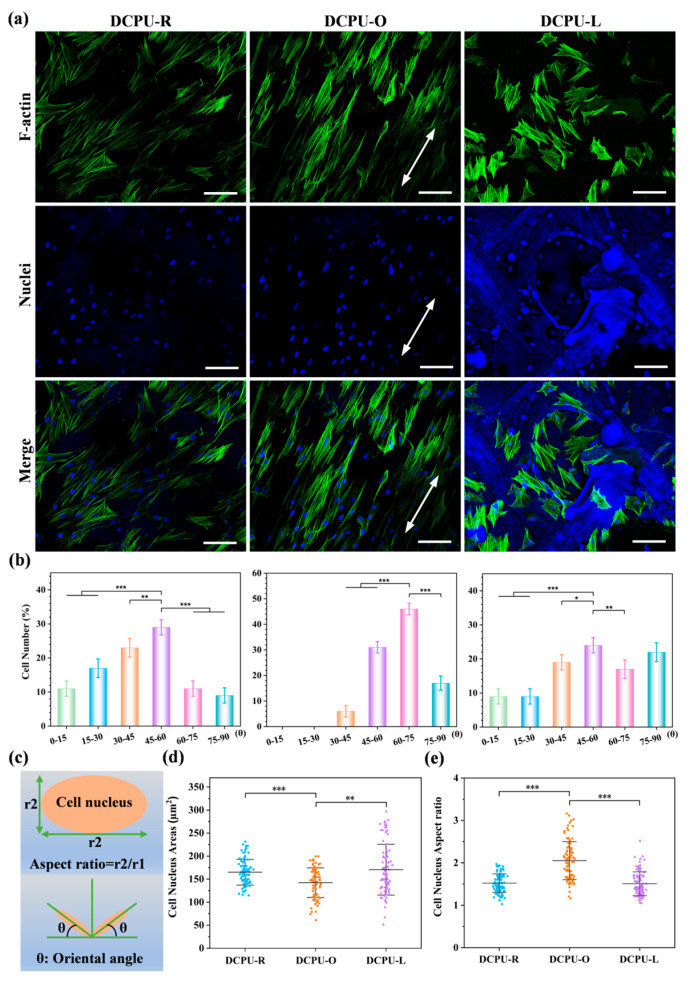
The morphology of BMSCs on DCPU networks with different surface topological structures: (**a**) fluorescence microscopy images of BMSCs on DCPU networks with various surface topological structure for 4 days. Green: F-actin; blue: cell nucleus. Scale bar: 100 μm. Arrows: cell orientation; (**b**) semi-quantitative analysis of BMSCs orientation angle on different samples; (**c**) the definition of aspect ratio for cells’ nucleus and orientation angle; (**d**) cell nucleus spreading area of BMSCs on different samples; (**e**) cell nucleus aspect ratio of various samples. (**b**,**d**,**e**) * *p* < 0.05, ** *p* < 0.01, *** *p* < 0.001 (*n* = 100).

Herein, the effect of topological surfaces on cell behavior was further investigated by observing the microscopic morphology of cells on DCPU fibrous scaffolds. As shown in Figure 6a, cells on DCPU samples with different topological surfaces attached and extended well, showing a spindle-shaped morphology. Specifically, the cells in the DCPU-O group grew along the fiber direction (arrows), thus showing a single direction. The cells in the DCPU-L group were mainly distributed in the ridge and center of mesh fibers. While the cells in the DCPU-R group were randomly distributed. Notably, the background color of the DCPU-L group was deeper, which was caused by the adsorption of dye. This phenomenon was consistent with the results of the highest water uptake content of the DCPU-L scaffolds (Figure S5b). In addition, semi-quantitative analysis of nucleus angles showed a significant orientation of cells in the DCPU-O group but not in the other two groups (Figure 6b).

Literally, cell morphology is closely related to its fate on scaffolds. Generally, cell senescence and differentiation can be preliminarily judged by the size and shape of the nucleus [63,64]. Senescent MSCs were characterized by enlarged, irregular morphology, followed by a decrease in phenotype and differentiation abilities [65]. Semi-quantitative data on nuclear morphology are shown in Figure 6d,e. Compared with the DCPU-R and DCPU-L groups, the DCPU-O group had a decreased nuclear area and an increased aspect ratio, meaning that cells on the DPCU-O fibrous scaffold exhibited better cell morphology and might have better differentiation potential.

The following two factors might contribute to this phenomenon. It has been reported that structures with a high surface area-to-volume ratio favored cell attachment and proliferation [66,67,68]. The specific surface area of the DCPU-O and DCPU-L scaffolds was higher than that of the DCPU-R scaffolds (Table S3), which provided an extracellular environment similar to that of native tissue to promote cell proliferation. On the other hand, scaffolds with higher conductivity could improve cell-to-cell communication and increase cell attachment and proliferation [69]. Herein, the results of EIS demonstrated that the oriented alignment endowed the DCPU-O scaffolds with the highest conductivity. Based on this, the cells on the surface of the DCPU-O scaffolds would receive stronger intercellular communication, which in turn promoted proliferation efficaciously.

#### 3.6.3. Osteogenic Differentiation with or without ES

**Figure 7:**
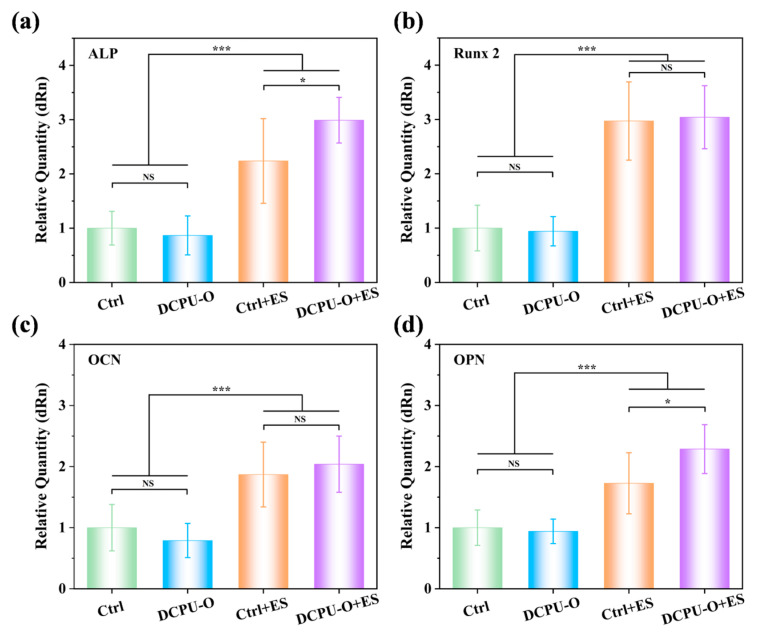
The expression of osteogenic related gene of BMSCs on DCPU-O fibrous networks with and without ES for 7 days: (**a**) ALP, (**b**) Runx 2, (**c**) OCN, (**d**) OPN. * *p* < 0.05, *** *p* < 0.001 (*n* = 3).

The above experiments have confirmed that the DCPU-O fibrous scaffolds had a stronger ability to promote cell proliferation and adhesion. On this basis, electrical stimulation was introduced to further explore the response of DCPU-O fibrous scaffolds to electrical signals. Herein, four typical osteogenic markers (ALP, Runx 2, OCN and OPN) were selected to evaluate osteogenic differentiation. As shown in Figure 7, in general, there was no significant difference in the expression of the four markers between the Ctrl and DCPU-O groups without ES. All four markers were significantly up-regulated in both the Ctrl and DCPU-O groups in the presence of ES, indicating that ES directly promoted osteogenic differentiation. Among them, ALP and Runx 2 are the key indicators regulating the early osteogenic differentiation [70]. As shown in Figure 7a,b, the DCPU-O group had a certain increase in ALP and Runx 2 expression in the presence of ES compared with the Ctrl group. Especially, the ALP expression of the DCPU-O + ES was notably increased by 1.33-fold than that in the Ctrl + ES group (*p* < 0.05). Moreover, OCN and OPN are markers of late osteogenic differentiation [71,72]. Similarly, the expression of OCN and OPN (*p* < 0.05) was elevated in the DCPU-O group with ES compared with the Ctrl group (Figure 7c,d). Specifically, the OPN expression was significantly up-regulated for DCPU-O + ES compared to the other three groups (*p* < 0.05), especially for a notable increase of 1.32-fold higher than that in the Ctrl + ES group. These results indicated that the DCPU-O fibrous scaffolds could respond to and amplify ES signals, thereby promoting osteogenic differentiation.

Exogenous electrical stimulation can directly act on cells or tissues to promote osteogenic differentiation and tissue regeneration [73]. However, the application of exogenous electrical stimulation has many limitations. Most importantly, it lacks selectivity of target cell/tissue, causing damage to non-target sites [74]. Conductive materials can provide substrates for cell/tissue adhesion and transmit ES signals, thereby improving targeting efficiency. The above results suggested that the DCPU-O scaffolds could respond to ES signals and improve the efficiency of ES signal transmission, which in turn enhanced osteogenic differentiation.

## 4. Conclusions

A series of conductive aniline trimer-based polyurethane fibrous scaffolds with different surface topological structures were successfully fabricated by using electrospinning. These fibrous scaffolds with three patterns exhibited tunable hydrophilicity, swelling capacity, and biodegradability. Moreover, they showed adjustable stress and elongation by changing the surface’s topological structure. Most importantly, the conductivity of the DCPU fibrous scaffolds transformed due to the difference in filament distribution, in which oriented DCPU scaffolds performed the highest conductivity compared to the other two patterns of scaffolds and casting membranes. Furthermore, all DCPU fibrous scaffolds showed good cytocompatibility better than AT-free DPU scaffolds. Meanwhile, the DCPU-O scaffolds exhibited the superior ability to promote BMSCs’ proliferation compared to DCPU-R and DCPU-L. Notably, the DCPU-O fibrous scaffolds exhibited significant positive effects on synergistically promoting the osteogenic differentiation of BMSCs when combined with ES. On this foundation, the DCPU-O fibrous scaffold could be a great candidate for tissue engineering applications with the combining of their unique topological structure and excellent electrical signal transfer property.

## Figures and Tables

**Table 1:** The summary of copolymers’ solubility in different solvents.

Solvent	HFIP	DMF	THF	Ethanol	Acetone	CH_2_Cl_2_
DPU	HD	HD	HD	HD	HD	D
DCPU	HD	HD	HD	D	D	D
PUAT	S	S	S	S	S	S

HD: highly dissolved (up to a concentration of at least 20 g/100 mL, *w*/*v*); D: dissolved (up to a concentration of at least 10 g/100 mL, *w*/*v*); S: swelled.

**Table 2:** Conductivity of DCPU fibrous scaffolds.

Sample	Conductivity (S/cm)	
	Dry State	Wet State
DCPU-R	4.02 ± 0.75 × 10^−10 b^	2.00 ± 0.10 × 10^−5 b^
DCPU-O	7.15 ± 0.49 × 10^−10^	4.09 ± 0.51 × 10^−5^
DCPU-L	3.36 ± 0.14 × 10^−10 a,b^	1.89 ± 0.15 × 10^−5 b^

^a^*p* < 0.05 versus DCPU-R group; ^b^ *p* < 0.001 versus DCPU-O group (*n* = 5).

**Table 3:** Model fit parameters from Nyquist plots.

Sample	*R*_i_ (Ω)	*Q*_dl_ (F/s)	*α*_dl_	*R*_e_ (Ω)	*Q*_g_ (Ω)	*α*_g_
DCPU-R	7014	1.18 × 10^−3^	1	6418	9.45 × 10^–5^	0.78
DCPU-O	3894	1.85 × 10^−3^	0.95	3520	9.20 × 10^–5^	0.80
DCPU-L	8671	1.06 × 10^–3^	0.97	6451	1.05 × 10^–5^	0.78

*Q* represents the peudocapacitance value and α represents the deviation from ideal capacitive behavior.

## Data Availability

The data presented in this study are available upon request from the corresponding author.

## Supplementary Materials

The following supporting information can be downloaded at https://www.mdpi.com/article/10.3390/jfb14040185/s1: Figure S1: Electrical stimulus (ES) apparatus; Table S1: Sequences of primers for the real-time PCR; Figure S2: ^1^H NMR spectrum of the synthesized AT (left) and fully reduced state AT (right); Figure S3: MS spectra of aniline trimer; Figure S4: (a,b) The thermal properties of DPU and DCPU copolymers. (a) TGA curves, (b) DSC curves. (c) X-ray diffraction curves of DPU and DCPU copolymers. (d) Molecular structure of the DCPU copolymer at various oxidation states; Figure S5: Physicochemical performances of the fibrous scaffolds. (a) Water contact angles. * *p* < 0.05, ** *p* < 0.01, *** *p* < 0.001 statistically significant compared with DCPU-O. (b) Water uptake content within 5 h. (c) Representative stress−strain curves. (d) Hysteresis loops under 5 cyclic tensile tests (a–c: *n* = 5); Table S2: Summary of the molar ratio, molecular weight, and polydispersion index of the DPU copolymer, DCPU copolymer, and PUAT organogel; Table S3: The summary of DCPU scaffolds’ specific surface area, pore volume, and pore size; Table S4: The mechanical properties of DPU-R and three kinds of DCPU fibrous scaffolds.
