# Supplementary material for: Synergetic Effect of Electrical and Topographical Cues in Aniline Trimer-Based Polyurethane Fibrous Scaffolds on Tissue Regeneration
Source: J Funct Biomater. 2023 Mar 28;14(4):185. doi: 10.3390/jfb14040185 (PMC10146274; doi:10.3390/jfb14040185)
Supplement: Supplementary file 1 [file jfb-14-00185-s001.zip › jfb-2308285-supplementary.pdf]

Supporting information

Synergetic Effect of Electrical and Topographical Cues in Aniline Trimer-based Polyurethane Fibrous Scaffolds on Tissue Regeneration

Yinglong Zhang [1], *Jiajing Tang* [1], *Wei Fang* [2], *Qing Zhao* [1], *Xiaoyu Lei* [1], *Jinzheng Zhang* [1], *Jieqiong Chen* [1], *Yubao Li* [1], *and Yi Zuo* [1], *

1. Research Center for Nano-Biomaterials, Analytical and Testing Center, Sichuan University, Chengdu, 610064, China
2. MOE Key Laboratory of Low-grade Energy, Utilization Technologies and Systems, CQU-NUS Renewable, Energy Materials & Devices Joint Laboratory, School of Energy & Power Engineering, Chongqing University, Chongqing, 400044, China
*Corresponding author: Prof. Yi Zuo

*E-mail address: zoae@scu.edu.cn（Yi Zuo）

Tel.: +86 28 85418178

Fax: +86 28 85417273

Mailing address: Analytical & Testing Center, Sichuan University, Chengdu 610064, People’s Republic of China

Materials and methods

1. Physicochemical characterizations of copolymers

^{1}H NMR: The ^{1}H NMR (400 MHz) spectra of AT and DCPU were obtained by a Bruker Ascend 400 MHz NMR instrument with the solution of DMSO-d_6. This test was carried out at room temperature and DMSO-d_6 as internal standard (δ 2.50 ppm).

FT-IR: FT-IR spectra of AT, L-Lysine, PCL, and copolymers DPU, DCPU were monitored on a Nicolet 6700 FT-IR spectrometer (Thermo Scientific Instrument) in the range of 3700-700 cm^{-1}. The spectra were taken as the average of 32 scans at a resolution of 4 cm^{-1}.

Gel permeation chromatography: GPC measurements were conducted at 40 °C by using the HLC-8320GPC (Tosoh, Japan). DMF was chosen as eluent and the flow rate was 1 mL/min to determine the average molecular weight and polydispersity index (PDI) of DPU and DCPU copolymers. Polystyrene standards (Shodex SM-105) were used as calibration of molecular weight standard curve.

Thermal properties: A thermogravimetric analysis (TGA2; Mettler Toledo, Switzerland) was used to determine the thermal stability of the DPU and DCPU copolymers under N_2 atmosphere with a flow rate of 10 mL min^{-1}. The scan range was from room temperature to 600 °C at the heating rate of 10 °C min^{-1}. A differential scanning calorimeter (DSC; TA Q250, TA Instrument) was used to study the glass transition of DPU and DCPU copolymers under nitrogen atmosphere at a flow rate of 10 mL min^{-1}.The copolymer was firstly heated from room temperature to 100 ℃ and equilibrated for 3 min to remove the thermal history, and then cooled down to -70 °C and heated to 100 °C again. The glass transition temperature (T_g) was analyzed and obtained from the second heating curve employing TA analysis software. An X-ray diffractometer (XRD; PANalytical Empyrean, Netherlands) was used to evaluate the crystallographic changes in the DPU and DCPU copolymers. Data was obtained in the 2θ range of 10°-50° at a scan rate of 0.05°/min.

2. Physicochemical performance of different scaffolds

Hydrophilicity and swelling capacity: The water wettability of scaffolds was measured by a water contact angle measurements (JY-82B, Chengde, China). The scaffolds were cut into circle (10 × 10 mm^2, ~200 μm thick). The swelling capacity of the scaffolds was studied by testing the water uptake profile of the scaffolds in PBS (pH=7.2-7.4) at 37 °C. At regular time intervals, the scaffolds were taken out of PBS. The water of the scaffolds' surface was removed by filter paper and the weight of them was recorded subsequently. The water uptake (%) of the scaffolds was calculated as:

$$\text{Water Uptake (\%)} = W_1\text{-}W_0/W_0 \times 100\% \qquad (1)$$

where W_1 represented the weight of wet scaffolds, and the W_0 represented the weight of initial scaffolds.

Mechanical testing: The mechanical properties of the DPU and DCPU scaffolds were evaluated by the uniaxial tensile test and cyclic tensile test employing a universal testing machine (SHIMADZU, AG-IC 50KN, Japan) equipped with a 500 N load cell. The sample was cut into dumbbell with a size of 4×50 mm. For uniaxial tensile test, the tensile test was conducted at a strain rate of 50 mm/min until specimen failure at room temperature. The initial modulus of scaffolds was calculated from the slope of stress-strain curve between the strain of 1% and 5%. The tensile strength and modulus data were both obtained by averaging over five specimens. For the cyclic tensile test, both loading and unloading cycles were performed at a constant jog rate of 15 mm/min with the maximum strain of 200% at room temperature.

Conductivity and electrochemical properties: A Keithley 6517A digital 4-probe tester with a linear probe head (1.0 mm space) was used to measure the electrical conductivity of the DCPU scaffolds under both dry and wet states. The scaffolds were swollen in PBS before the wet state conductivity test. The electrical conductivity was calculated as the following equation:

$$\delta = 1/\rho \qquad (2)$$

where δ represents conductivity and ρ represents the resistance.

The electrochemical impedance spectroscopy (EIS) measurements of the DCPU scaffolds were carried out by using a CHI 600E electrochemical work-station (CH Instruments) in an electrochemical cell installed with Pt sheet as counter electrode and Ag/AgCl as a reference electrode. The DCPU scaffolds was clamped on Pt sheet as working electrode. The frequency range between 0.1 and 100 kHz was scanned in 1M HCl with an applied bias of 0.01 V vs.

Ag/AgCl. The EIS data of the DCPU scaffolds were fitted by using an equivalent circuit model for further analysis.

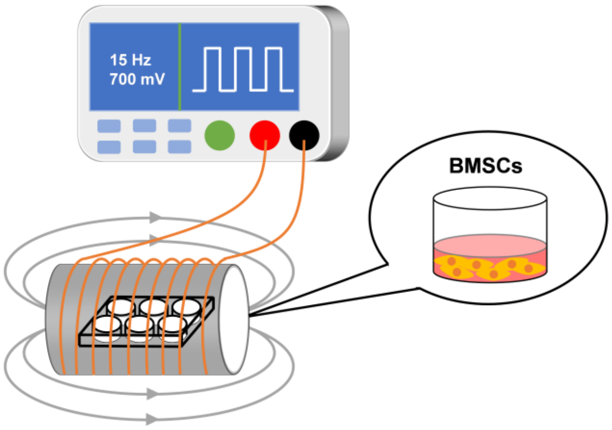

Figure S1. Electrical stimulus (ES) apparatus.

Table S1. Sequences of primers for the real-time PCR.

Gene	Forward primer sequence	Reverse primer sequence
ALP	5-AGGGCTCCGGTTACTAAA-3’	5-GACCACAGCACCTACAATAC-3’
Runx 2	5-CAAGAAGGCACAGACAGAA-3’	5-GGGACACCTACTCTCATACT-3’
OCN	5-GGACCCTCTCTCTGCTCACTCTG-3’	5-ACCTTACTGCCCTCCTGCTTGG-3’
OPN	5-GACGATGATGACGACGACGATGAC-3’	5-GTGTGCTGGCAGTGAAGGACTC-3’
GAPDH	5-ACAGCAACAGGGTGGTGGAC-3’	5-TTTGAGGGTGCAGCGAACTT-3’

Results and Discussion

1. Synthesis of AT

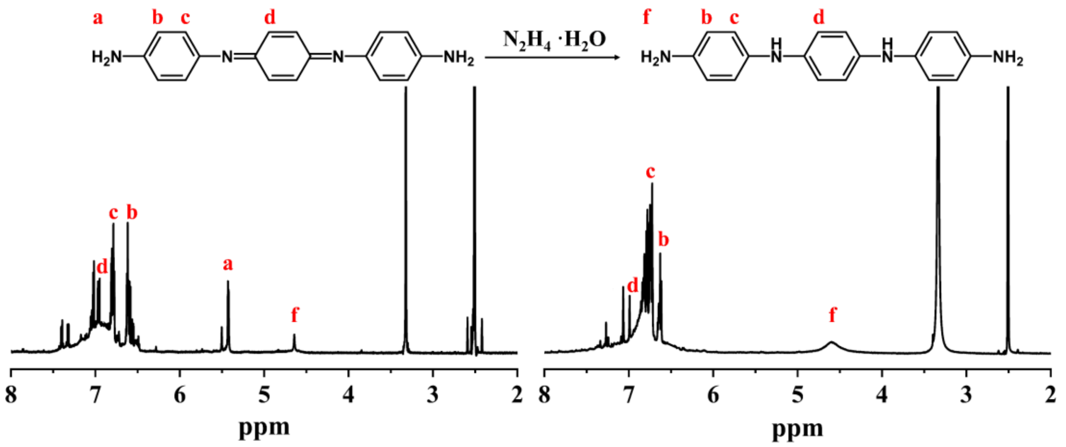

Figure S2. ^{1}H NMR spectrum of the synthesized AT (left) and fully reduced state AT (right).

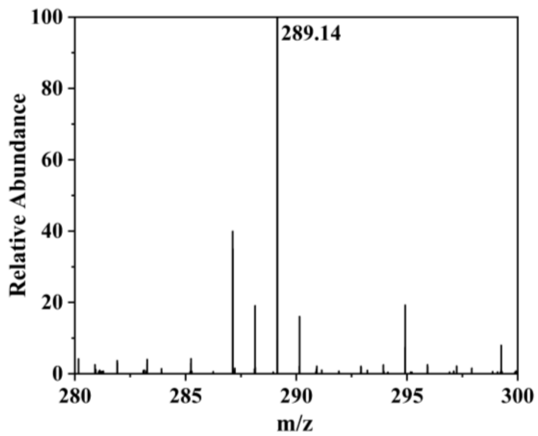

Figure S3. MS spectra of aniline trimer.

2. Thermal properties of DPU and DCPU copolymers

The thermal properties of DPU and DCPU copolymers were measured by TGA and DSC analysis (Figure S4a-b). The decomposition temperatures (T_d) rose from 326.3 °C of DPU to 384.3 °C of DCPU, indicating that the AT segment largely increased the decomposition temperature of DCPU copolymer. On a deeper level, it improved the thermal stability of DCPU copolymer. Moreover, the glass transition temperature (T_g) of DCPU (-56.1 °C) was higher than that of DPU copolymer (-59 °C), which might cause by the decreased crystallinity of the copolymer, revealing that there were more hard segments dispersed in the soft phase and more robust interaction between the polymer chains in DCPU copolymer.[1, 2] This result was

confirmed by XRD (Figure S4c). Two characteristic crystal peaks at $2\theta = 21.3°$ and 23.4° were detected in the X-ray diffraction curves of the DPU copolymer, which were attributed to PCL segment. However, there was a broad peak in the XRD curves of DCPU, which was a typical pattern of materials with a low degree of crystallinity.

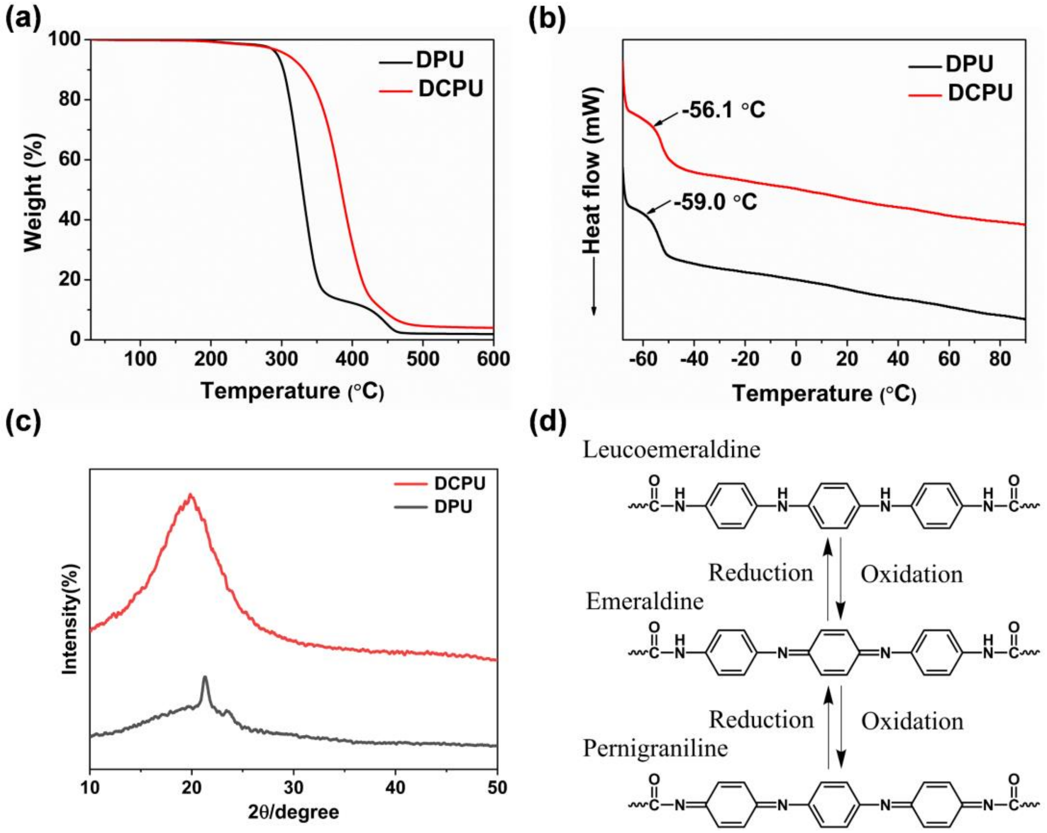

Figure S4. (a-b) The thermal properties of DPU and DCPU copolymers. (a) TGA curves, (b) DSC curves. (c) X-ray diffraction curves of DPU and DCPU copolymers. (d) Molecular structure of the DCPU copolymer at various oxidation states.

3. Physicochemical performance of different scaffolds

3.1. Hydrophilicity and swelling capacity

The water contact angle values of DPU and DCPU fibrous scaffolds were shown in Figure S5a. The DCPU-L fibrous scaffold presented the lowest hydrophilicity with a water contact angle of 85°, and DCPU-R scaffold showed the contact angle of 72°, whereas a contact angle of 65° was for DPU-R scaffold without hydrophobic AT added. Interestingly, the DCPU-O scaffold showed the highest hydrophilicity with a water contact angle of 63° in contrast to other AT-added scaffolds ($p < 0.01$ to DCPU-R & $p < 0.001$ to DCPU-L, respectively). When the water contacted the surface of the DCPU-O scaffold, it diffused along the direction of the

filament orientation rapidly until it gradually wetted the surface of the scaffold, which endowed the DCPU-O scaffold best hydrophilia.

The swelling capacity of the fibrous scaffolds was evaluated by the water uptake ratio soaked in phosphate buffer saline (PBS)within 5 h (Figure S5b), due to the cell adhesion behaviour was completed within 4-5 hours. The DCPU-R and DCPU-O fibrous scaffolds showed slightly lower water absorption compared to the DPU-R scaffold in early contact with water for its hydrophobic benzene ring of the added AT segment. However, the DCPU-L fibrous scaffold showed the best swelling capacity compared with other groups. This phenomenon was due to the highest specific surface area and large number of uneven grid cells on the surface of DCPU-L fibrous scaffold (Figure 3b & Table S3), which could hold more water. The controllable hydrophilicity and swelling capacity of the conductive fibrous scaffolds suggested that they could be employed as tissue engineering materials by changing the ratio of the added AT segment and/or the topological surface structure of fibrous scaffolds.

3.2. Mechanical properties

Mechanical properties of biomaterials play a critical role in tissue engineering applications.[3] Figure S5d presented the fibrous scaffolds' tensile stress–strain curves, and Table S4 showed the data of the mechanical properties of fibrous scaffolds tested at room temperature. The tensile strength of fibrous scaffolds increased significantly with the addition of AT compare to the pristine DPU-R scaffolds. The DCPU-O scaffold showed the highest tensile strength due to their well-aligned arrangement among all scaffolds. On the other hand, the tensile strengths of DCPU-R and DCPU-L scaffolds were close which were significantly lower than that of DCPU-O scaffolds ($p < 0.05$). The influent factor on mechanical properties of these scaffolds was mainly based on the anisotropy of fiber distribution but not the fibrous diameter (Figure 3b & c), even the diameter sizes of DCPU-R and DCPU-L scaffolds were larger than that of DCPU-O scaffolds ($p < 0.001$).

The elongations at break of all fibrous scaffolds were above 400%, exhibiting superior space expansibility (Table S4). In the stretching process, the fibers in the DCPU-R scaffolds could gradually move from the random arrangement to the directed arrangement along with the tension orientation, thus showing a higher elongation at break ($p < 0.05$). Conversely, the knitting nodes in the DCPU-L scaffolds caused by the fiber intersection limited the fiber movement, so its elongation at break was low. Furthermore, the elongations at break of DCPU-O group were significantly lower than that of DCPU-R and DCPU-L fibrous scaffolds, which was due to the second stretching effects when the aligned filaments were collected by the high-speed receiving drum ($p < 0.05$).[4] The cyclic stretching of DPU-R and DCPU fibrous scaffolds

at a maximum strain of 200% was done to detect their resiliency shown in Figure S5e. The hysteresis loop areas changed relatively little during the first stretch and subsequent stretch cycles. All fibrous scaffolds presented small irreversible deformations at a maximum strain of 200% so that the stretchability of these scaffolds is good for periosteum repairing.

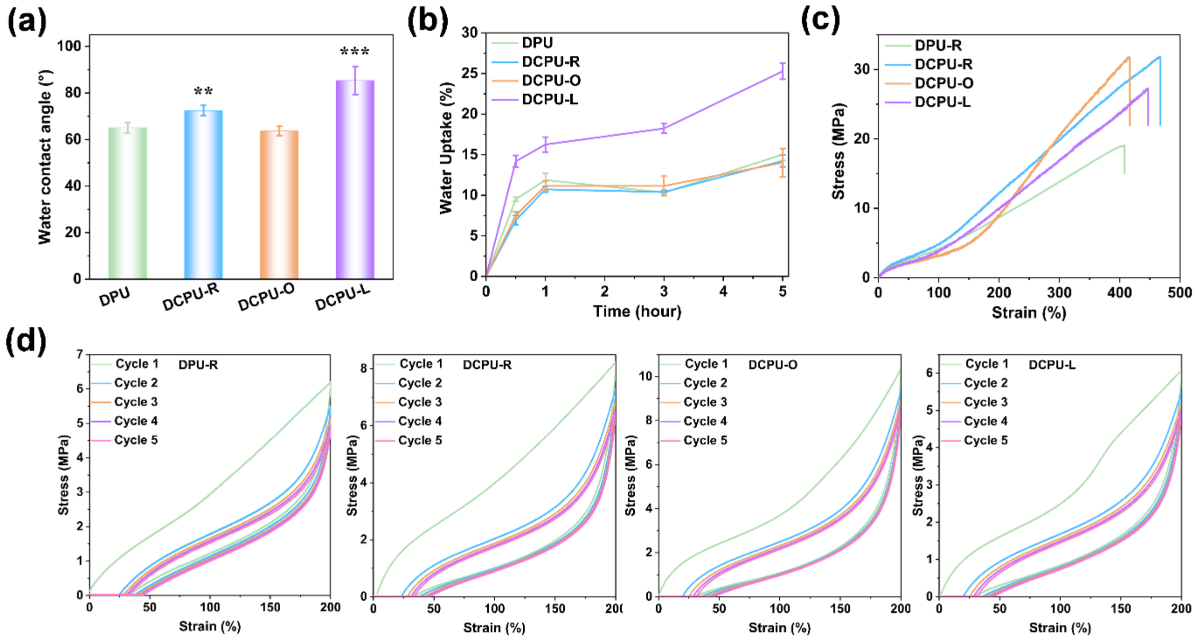

Figure S5. Physicochemical performances of the fibrous scaffolds. (a) Water contact angles. **p* < 0.05, ***p* < 0.01, ****p* < 0.001 statistically significant compared with DCPU-O. (b) Water uptake content within 5 h. (c) Representative stress–strain curves. (d) Hysteresis loops under 5 cyclic tensile tests. (a-c: *n*=5)

Table S2. Summary of the molar ratio, molecular weight, and polydispersion index of the DPU copolymer, DCPU copolymer, and PUAT organogel.

**Sample**	**Raw material ratio of PCL:IPDI:L-Lysine:AT**	**AT ratio (g/g)**	***Mn* (g/mol)**	**PDI**
DPU	1:2:1:0	0	25835	2.00
DCPU	1:2:0.5:0.5	5	33088	2.38
PUAT	1:2:0.5:0.5	5	29772	2.11

Table S3. The summary of DCPU scaffolds' specific surface area, pore volume, and pore size.

Sample	Specific Surface Area (m^2/g)	Average Pore Volume (mm^3/g)	Average Pore Size (nm)
DCPU-R	0.46	0.33	3.67
DCPU-O	0.91	0.70	3.88
DCPU-L	1.43	1.70	6.83

Table S4. The mechanical properties of DPU-R and three kinds of DCPU fibrous scaffolds.

Sample	Stress (MPa)	Strain (%)	Initial modulus (MPa)
DPU-R	20.31±3.72	433.05±24.46	6.35±1.23
DCPU-R	29.72±5.01$^{b, d}$	468.99±43.43$^{a, e}$	11.59±1.99$^{c, d}$
DCPU-O	32.37±4.08^{c}	413.78±15.53	13.41±2.39^{c}
DCPU-L	29.80±1.95$^{b, d}$	447.34±18.08^{d}	10.92±3.30$^{c, e}$

$^{a}p < 0.05$, $^{b}p < 0.01$, $^{c}p < 0.001$ versus DPU-R group; $^{d}p < 0.05$, $^{e}p < 0.01$ versus DCPU-O group. (n=5).

Stretchability of DCPU scaffolds had no effect on electrical properties, the difference of electrical property among three kinds of DCPU scaffolds was mainly affected by the topological structure.

References

[1] M. Deng, J. Wu, C. A. Reinhart-King, C.-C. Chu, *Acta Biomaterialia* **2011**, *7*, 1504.
[2] X. Wei, K. Bagdi, L. Ren, P. Shah, K. Seethamraju, R. Faust, *Polymer* **2013**, *54*, 1647.
[3] J. Chen, B. Guo, T. W. Eyster, P. X. Ma, *Chemistry of Materials* **2015**, *27*, 5668.
[4] S. Shao, S. Zhou, L. Li, J. Li, C. Luo, J. Wang, X. Li, J. Weng, *Biomaterials* **2011**, *32*, 2821.
